# Validation of an Electronic Visual Analog Scale App for Pain Evaluation in Children and Adolescents With Symptomatic Hypermobility: Cross-sectional Study

**DOI:** 10.2196/41930

**Published:** 2022-10-26

**Authors:** Muhammad Maarj, Verity Pacey, Louise Tofts, Matthew Clapham, Xavier Gironès Garcia, Andrea Coda

**Affiliations:** 1 School of Health Sciences, College of Health, Medicine and Wellbeing University of Newcastle Ourimbah Australia; 2 Department of Health Sciences Macquarie University Sydney Australia; 3 Hunter Medical Research Institute Equity in Health and Wellbeing Research Program New Lambton Heights, New South Wales Australia; 4 Facultat de Ciències de la Salut de Manresa Universitat de Vic-Universitat Central de Catalunya Manresa Spain

**Keywords:** hypermobility syndrome, Ehlers-Danlos syndrome, hypermobility, hypermobile, mobile application, mobile app, pain measurement, pain, validation, validate, scale, measure, pain severity, pediatric, validation, visual analogue scale, mHealth, mobile health, mobile app, children, adolescent, youth, child, digital health tool

## Abstract

**Background:**

Rapid advances in mobile apps for clinical data collection for pain evaluation have resulted in more efficient data handling and analysis than traditional paper-based approaches. As paper-based visual analogue scale (p-VAS) scores are commonly used to assess pain levels, new emerging apps need to be validated prior to clinical application with symptomatic children and adolescents.

**Objective:**

This study aimed to assess the validity and reliability of an electronic visual analogue scale (e-VAS) method via a mobile health (mHealth) App in children and adolescents diagnosed with hypermobility spectrum disorder/hypermobile Ehlers-Danlos syndrome (HSD/HEDS) in comparison with the traditional p-VAS.

**Methods:**

Children diagnosed with HSD/HEDS aged 5-18 years were recruited from a sports medicine center in Sydney (New South Wales, Australia). Consenting participants assigned in random order to the e-VAS and p-VAS platforms were asked to indicate their current lower limb pain level and completed pain assessment e-VAS or p-VAS at one time point. Instrument agreement between the 2 methods was determined from the intraclass correlation coefficient (ICC) and through Bland–Altman analysis.

**Results:**

In total, 43 children with HSD/HEDS aged 11 (SD 3.8) years were recruited and completed this study. The difference between the 2 VAS platforms of median values was 0.20. Bland–Altman analysis revealed a difference of 0.19 (SD 0.95) with limits of agreement ranging –1.67 to 2.04. An ICC of 0.87 (95% CI 0.78-0.93) indicated good reliability.

**Conclusions:**

These findings suggest that the e-VAS mHealth App is a validated tool and a feasible method of collecting pain recording scores when compared with the traditional paper format in children and adolescents with HSD/HEDS. The e-VAS App can be reliably used for pediatric pain evaluation, and it could potentially be introduced into daily clinical practice to improve real-time symptom monitoring. Further research is warranted to investigate the usage of the app for remote support in real clinical settings.

## Introduction

Reliable and validated assessment tools of pain intensity are required to evaluate and implement appropriate and timely therapies. In recent years, digital health advances have led to significant progression in real-time pain-related data collection that may improve pain management [1-4]. A recent meta-analysis of 7977 children and adults reported that pain-related data collected by electronic devices that measured pain intensity mainly using a visual analog scale (VAS) showed equal to or greater reliability than traditional paper collection methods [1]. Furthermore, the study found that patients preferred using the electronic format of data collection to the paper version [1].

The current widely used method to evaluate pain intensity is the VAS instrument, which has been used in clinical and research settings for a number of years to record self-reported pain levels in both adults and children [1,5-7]. This approach is shown to have moderate reliability in children over 5 years of age [8] and validated in children 7 years of age and over [7,9]. Typically, the VAS is a 10-cm–long premeasured horizontal line anchored at either end representing subjective feeling by the extremes of pain level with 0 mm marked as “no pain at all” to 10 cm rated as the “worst possible pain” [6]. Traditionally, the VAS is completed in a paper-based format. Despite the accuracy and extensive clinical application of the paper version, there are a number of limitations of the paper-based VAS (p-VAS), including incomplete or incorrect marking limiting validity of data, inefficient and extensive data handling by clinicians and researchers, and manual processing for each patient with the possibility of introducing error during data measurement and entry [3]. In contrast, an electronic VAS (e-VAS) allows for automatic calculation of the VAS score, preventing possible human errors when using a ruler.

To overcome these potential barriers of the p-VAS version, Escalona-Marfil et al [10] recently developed a novel e-VAS to measure pain level through an “Interactive Clinics” app, which has been since validated for use in healthy children [11]. The electronic VAS method allows the collection of real-time data from patients and direct integration with electronic health records, reducing burden on clinicians and researchers. Furthermore, support for efficient, valid, and reliable approaches in timely assessment of pain severity is critical for evaluating the effectiveness of pain therapies and implementation of early interventions in the pediatric population. Despite emerging evidence on psychometric validation of the digital VAS versions in pediatrics [3,10-13], the feasibility of the application of the e-VAS in children with symptomatic hypermobility conditions has not been reported. Generalized joint hypermobility (GJH) is a connective tissue condition characterized by an excessive range of motions that affects multiple joints [14]. Almost 1 in 5 children with GJH experiences symptoms [15,16], particularly chronic pain [17], with a negative impact on their quality of life [18,19]. Once a young person with GJH has musculoskeletal pain or other symptoms, a diagnosis of hypermobility spectrum disorder/hypermobile Ehlers-Danlos syndrome (HSD/HEDS) is usually made.

This mobile Health (mHealth) tool might prove beneficial for patients living in geographically remote areas, where access to specialists is limited. Patients and parents or caregivers may not always be required to visit the hospital, consequently saving the time and money required to travel long distances from rural areas. Furthermore, health professionals can access the recorded pain-related information digitally without the need to contact the patient. If introduced within different clinics that provide care to children and adolescents affected by HSD/HEDS, the e-VAS can support early pain detection, preventing incidences of unnecessary prolonged pain with a consequent improvement in the patient’s quality of life. This possible digital health advancement in pediatric pain management may also lead to a reduction in absenteeism from school. The aim of this study was to determine the validity and feasibility of a newly developed e-VAS app interface in recording pain intensity in children and adolescents with symptomatic hypermobility.

## Methods

### Study Design

A cross-sectional study design was used to evaluate the validity and reliability of the e-VAS version for pain measurement in children with hypermobility.

### Ethics Approval

Ethics approval for this study (H-2020-0387) was granted by Human Research Ethics Committee of University of Newcastle (Callaghan, New South Wales, Australia).

### Settings and Participants

Participants were recruited from Narrabeen Sports and Exercise Medicine Centre (Narrabeen, New South Wales, Australia). Eligibility criteria included children and adolescents aged between 5 and 18 years and diagnosed with generalized joint hypermobility (Beighton score of ≥5 for adolescents in or post puberty and ≥6 before puberty) with sufficient English language and cognitive skills to rate the severity of pain. Participants were recruited if they reported lower limb pain of at least 2 out of 10 on the VAS assessment tool in the previous month.

Participants were excluded if they were diagnosed with major cognitive or psychiatric disorders that interfered with rating of pain severity and other medical conditions that may have contributed to chronic or recurrent pain or interfered with their ability to use their hand for documenting p-VAS scores.

Demographic data were collected, including age, sex, height, weight, and BMI. The Beighton Score [20] was used to measure joint hypermobility on a 9-point scale. To prevent bias, the same clinical researcher (MM) completed all data collection.

### Measuring Tools

**Figure 1:**
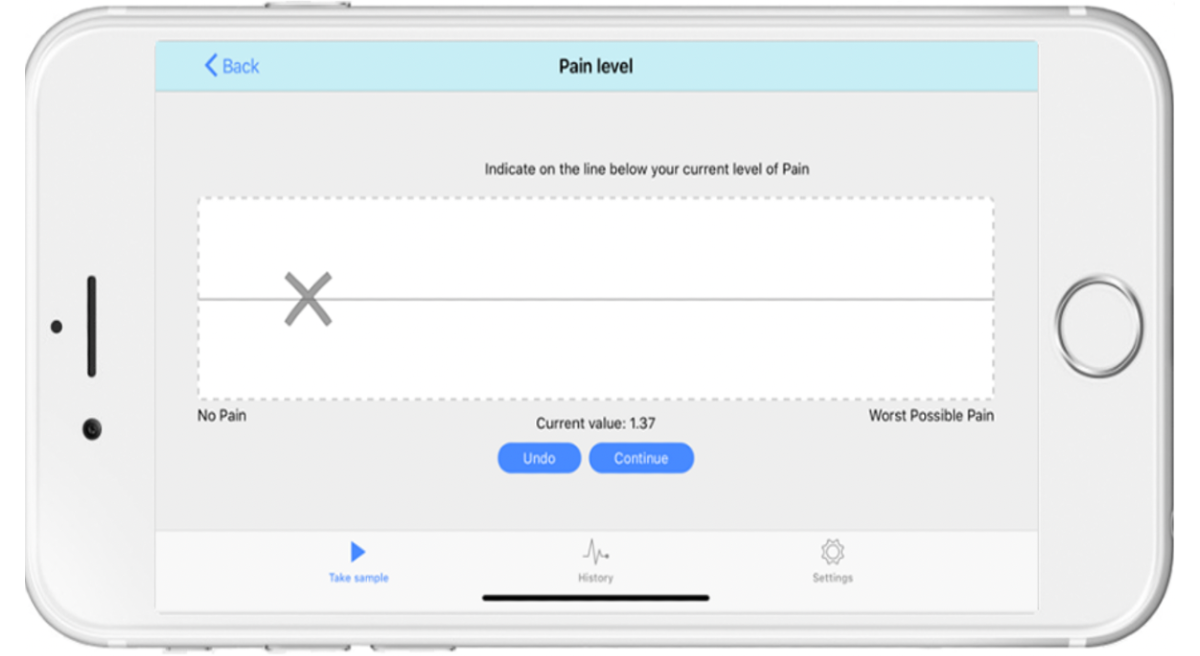
The electronic visual analog scale app technology used to score pain intensity.

Pain recording data were collected at one time point from each consenting participant using the e-VAS app (version 1.2.4, accessible to both iOS and Android devices, powered by Bit Genoma Digital Solutions Ltd), which was downloaded for free on either the parents’ or participants’ smartphones. In accordance with the digital health policy outlined by the European Pain Federation [21], e-VAS data collected on the App were safely stored on the country-based server (Australia). The e-VAS version displays a horizontal gray line on a white background (Figure 1). The traditional p-VAS format displays a 100-mm horizontal line. In both the e-VAS and p-VAS, the end point labels on the left and right sides of the horizontal line indicated “no pain” and “worst possible pain,” respectively.

### Procedure

At the initial appointment, each participant was asked to recall their pain experience during the past month. A researcher who was independent of recruitment and data collection (AC) created the randomization sequence in blocks of 10 each by using a freely available web-based number generator software. Allocation concealment was achieved by AC masking the sequence into consecutively numbered sealed and opaque envelopes. Sealed envelopes were strictly opened by the principal investigator (MM) only on the day of participant’s initial consultation to reveal the sequence of the e-VAS and p-VAS. All participants completed assessments on both VAS platforms.

Prior to data collection, a full demonstration was provided to the participant with an opportunity to ask questions. For the e-VAS recording of pain level, the patient’s smartphone was placed flat on a table, and each participant was asked to apply single-finger pressure on the horizontal line displayed on the touch screen and to indicate the location corresponding to the pain intensity experienced. The e-VAS mobile app automatically calculated the pain rating from collected results, which were then directly synchronized to the principal investigator’s project account on the Interactive Clinics web-based platform that was password protected, thus minimizing data handling and streamlining the processing of data extrapolation. Data from the paper version were extrapolated by the same investigator (MM) using a standard ruler, and results were manually entered into a spreadsheet for statistical analysis.

### Statistical Analysis

Descriptive statistics including median, minimum, and maximum as well as mean (SD) values for the e-VAS and p-VAS outcomes were calculated by an independent statistician. The statistician was blinded to both the allocation concealment (p-VAS and e-VAS) and the identity of the participants. All statistical analyses were performed using R (version 4.1.3; R Core Team) [22].

For construct validity and reliability of the e-VAS and agreement between the 2 VAS methods, exploratory Bland–Altman graph analysis and the intraclass correlation coefficient (ICC) were used, respectively [23,24]. For each participant, the difference between e-VAS and p-VAS measurements was plotted against the average of each method. The analysis was performed by calculating the limits of agreement as mean of the difference (SD 1.96) multiplied by the SD of the difference. For comparison, a nonparametric approach to the limits of agreement using 2.5 and 97.5 percentiles was included. For absolute agreement between e-VAS and p-VAS values, the ICC, ICC(3,1), or equivalently ICC(A,1) derived from a 2-way mixed-effects model was used. ICC values of >0.75 indicate good agreement [25]. 

## Results

### Participant Characteristics

A total of 43 children and adolescents diagnosed with HSD/HEDS participated in this study. Anthropometric and demographic characteristics at baseline are summarized in Table 1.

**Table 1:** Clinical and demographic characteristics of the study sample (N=43).

Characteristics of participants		Values		
**Gender, n (%)**				
	Female	28 (65)		
	Male	15 (35)		
Age (years), mean (SD)		11.0 (3.8)		
Hypermobility (Beighton score), mean (SD)		7.0 (1.3)		
Weight (kg), mean (SD)		40 (16)		
Height (m), mean (SD)		1.45 (0.2)		
BMI (kg/m^2^), mean (SD)		18.3 (3.5)		
**School education level, n (%)**				
	Primary school	28 (65)		
	Secondary school	15 (35)		

### Comparison Between the e-VAS and p-VAS Versions

The summary statistics for the 2 VAS platforms (e-VAS and p-VAS) are presented in Table 2. The difference between the 2 methods of median values is 0.20 among children and adolescents with symptomatic hypermobility.

**Table 2:** Summary of statistics for visual analog scale (VAS) assessments in children and adolescents with hypermobility spectrum disorder (N=43).

Instrument	Score	
	Median (IQR)	Mean (SD)
Electronic VAS	5.90 (1.40-9.50)	5.89 (1.99)
Paper-based VAS	5.70 (2.00-9.30)	5.70 (1.77)

**Figure 2:**
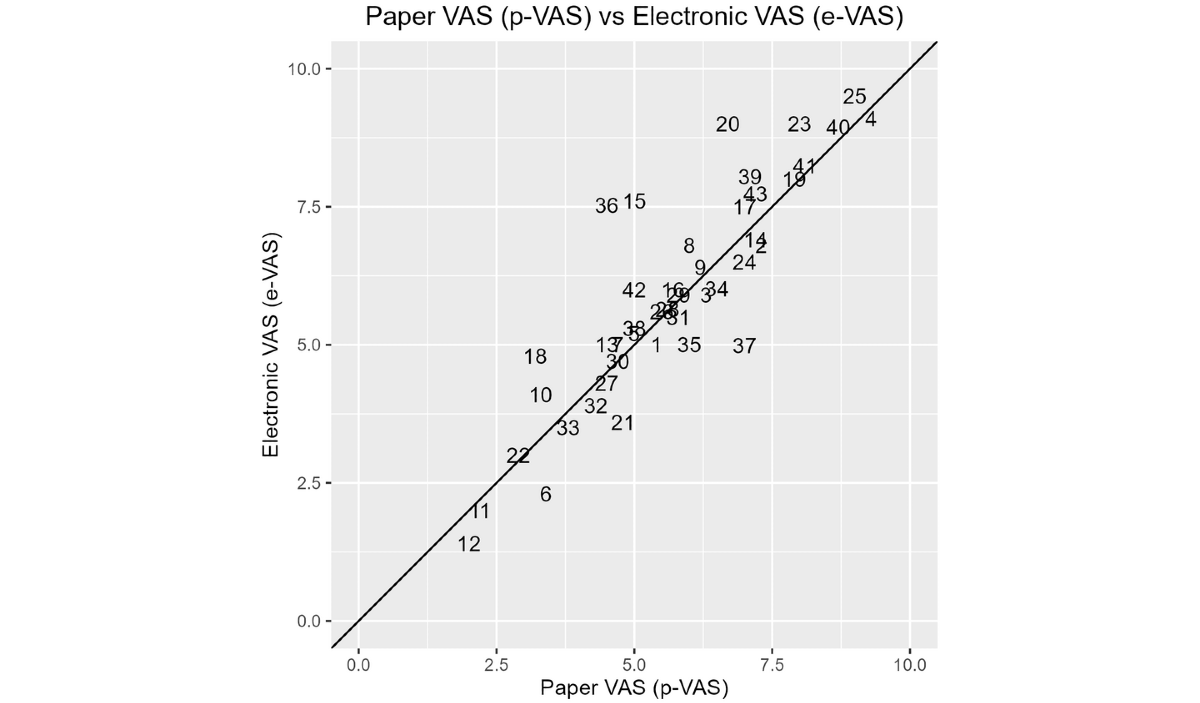
Scatter plot of data for the electronic visual analog scale (e-VAS) versus paper-based visual analog scale (p-VAS). Points on the graph indicate each participant. VAS: visual analog scale.

The scatter plot for the e-VAS compared to that of the p-VAS with a line of equality is presented in Figure 2 for every participant (numbered) with no apparent systematic difference between e-VAS and p-VAS methods. Points that lie on the diagonal line are in complete agreement between the 2 methods. The reliability estimated by ICC for baseline was 0.87 with a 95% CI of 0.78-0.93, indicating good agreement.

**Figure 3:**
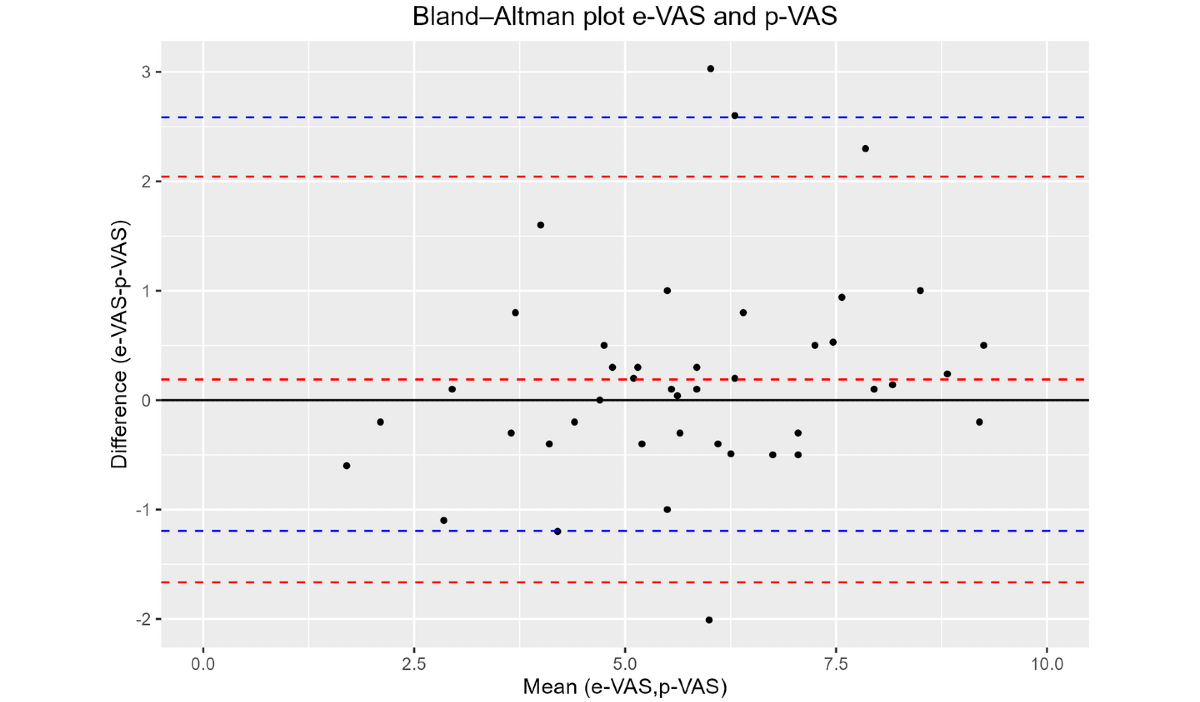
Bland-Altman plot for differences against the mean of scores on the electronic visual analog scale (e-VAS) and paper-based visual analog scale (p-VAS). Dashed red lines indicate the mean difference and limits of agreement. Blue dashed lines indicate the 2.5 and 97.5 percentiles of the difference. The solid black line is the zero reference for the difference.

The Bland–Altman plot is presented in Figure 3. The mean of the difference between e-VAS and p-VAS was 0.19 (SD 0.95) with limits of agreement ranging –1.67 to 2.04. The 2.5 and 97.5 percentiles of the difference were –1.19 and 2.58, respectively. There was a slight bias toward e-VAS with e-VAS measuring 0.19 higher on average than the p-VAS method.

## Discussion

### Principal Findings

To our knowledge, this is the first study that investigated the validity and reliability of an e-VAS in children and adolescents with HSD/HEDS for pain evaluation. Our results show that the e-VAS and the p-VAS can be used interchangeably. Instrument agreement was present between the p-VAS and e-VAS methods with good reliability (ICC=0.87) and validity (mean difference 0.19).

These findings are supported by previous reports of good reliability and validity of the e-VAS in healthy children, adolescents, and adult participants without pain on the newly designed Interactive Clinics app compared to that of the paper version [10,11]. In a prospective cross-sectional study, Escalona-Marfil et al [10] reported good reliability of the e-VAS method, as indicated by an ICC of 0.86 (95% CI 0.81-0.90) in healthy adults aged 18-65 years. In addition to evaluating pain in adults, Turnbull et al [11] reported that the e-VAS can be used interchangeability with the p-VAS in the pediatric population by showing moderate-to-good reliability with an ICC of 0.80 (95% CI 0.70-0.87) in healthy children and adolescents aged 10-18 years. Furthermore, there is strong consolidated evidence in support of the e-VAS’s comparability with the p-VAS version [26].

Advances in digital health have enabled emerging application of mHealth tools in pain management of children and adolescents by capturing real-time pain-related data, reducing recall bias, and improving responsiveness of health professionals [27]. The findings from a recent meta-analysis revealed a strong correlation between paper methods and electronic capture of pain-related outcomes with respect to completeness of patient-reported data collection, score equivalency, ease of use, and acceptability supporting their use in the clinical setting and in interventional research [1].

Other benefits of electronic data capture methods in the management of patients with pain have been reported to include a significant decrease in the severity of pain, worse pain, and an improved quality of life over time in both adult and adolescent patients (aged 12-68 years) who used a pain management app on a mobile device [28]. A recent meta-analysis of noncancer pain in adult patients further reported that app-based pain interventions were significantly more effective at reducing different types of chronic pain in comparison with control groups [29]. Furthermore, both patients and health care professionals prefer using pain Apps [28] with high compliance (83%) reported in adult patients (aged 19-65 years) completing electronic diaries for pain assessment [30].

Although there are other alternative instruments to the VAS, such as the numeric rating scale and verbal rating scale, the VAS has the greatest clinical utility, is in widespread clinical use, and has been the best measure of self-reported pain in children aged ≥7 years [31]. However, there are certain limitations of the p-VAS. For example, there is potential for drawing the line outside of the 0-10–point scale—or at an angle—and introducing human error while using a ruler [32], whereas the e-VAS allows for automatic calculation of the VAS score, thus preventing invalid responses and increasing consistency as the same measuring method is used, thereby reducing potential for error [33].

### Clinical Implications

The growing use of digital health has the potential to improve adherence to pain reporting [34,35], allow real-time data capture [35], and consequently improve communication between clinicians and their patients [36]. Novel mHealth tools, such as the e-VAS App, support efficient capture and recording of patient-reported outcome measures in day-to-day clinical practice, which improves clinicians’ insights into the effectiveness of any intervention they provide with the aim of reducing pain.

As part of the daily clinical management of pain in children with HSD/HEDS, the e-VAS app is a useful tool to record pain at a precise time and as frequently as needed. This, in turn, may improve the implementation of more appropriate and timely pain management strategies. The e-VAS app further allows health care professionals to record the time and day of assessment accurately with a lower chance of potential error during clinical data collection. In addition, completion of the VAS assessment is possible remotely as the data can be sent electronically to medical records, allowing for real-time tracking of pain and helping prevent a potential recall bias. Further clinical utility of these digital health advances needs to be explored in geographically remote areas with limited availability of allied health care professionals. Accordingly, further research is warranted to evaluate the efficacy and functional capabilities of these novel apps for clinical pain management in the pediatric population.

### Limitations and Strengths

A major methodological advantage of this study was the use of block randomization for the e-VAS and p-VAS sequences when collecting data from children and adolescents with HSD/HEDS. Further strengths of this study include comparison of the digital platform with paper-based assessment and statistical analyses.

The findings of this study need to be considered in light of some limitations. Data were collected from a single center, and two-thirds of the sample consisted of females, which might limit the generalizability of our findings to the whole pediatric population with HSD/HEDS. However, the sample size clearly reflects the higher prevalence of HSD in females [37]. Furthermore, it is important to note that there might be a possible recall bias, especially among the younger children relying on recalling pain intensity the month before. To reduce possible confounding factors, e-VAS and p-VAS recordings were undertaken at the same time, with a maximum gap of only 1 minute between the data collection and rating of pain intensity. While the use of the VAS is generally recommended in children aged ≥7 years, to increase the power for our study, we included children aged 5-6 years in this study since cognitive abilities are more reliable predictors than chronological age in effective use of the VAS [6]. In addition, use of the VAS in 5-6–year-old children has been found to show a moderate-to-strong correlation with the rating of pain intensity level further supporting the application of this instrument in younger children [38]. Therefore, future trials should also have an increased sample size to include the younger pediatric population.

### Conclusions

The findings of this study indicate that the e-VAS and p-VAS are interchangeable among children and adolescents diagnosed with HSD/HEDS. This study provides strong support for the clinical application of digital health in pain assessment in this pediatric population. The advancement in easily accessible digital health pain applications may have the potential to facilitate early clinical decision-making and to improve compliance with pain reporting. In conclusion, emerging digital health platforms may also promote better communication between clinicians and patients by providing more accurate and objective real-time monitoring of symptoms among children and adolescents with HSD/HEDS.

## Abbreviations

e-VAS: electronic visual analog scale
GJH: generalized joint hypermobility
HEDS: hypermobile Ehlers-Danlos syndrome
HSD: hypermobility spectrum disorder
ICC: intraclass correlation coefficient
mHealth: mobile health
p-VAS: paper-based visual analog scale
VAS: visual analog scale
